# Genome-Wide Analysis of the DC1 Domain Protein Gene Family in Tomatoes under Abiotic Stress

**DOI:** 10.3390/ijms242316994

**Published:** 2023-11-30

**Authors:** Guobin Li, Jiao Dang, Jiaqi Pan, Jingyi Liu, Tieli Peng, Guo Chen, Rongqun Wang, Songshen Hu, Xiaojing Li, Xiaohui Hu

**Affiliations:** 1 College of Horticulture, Northwest A&F University, Yangling 712100, China; liguobin@nwafu.edu.cn (G.L.); dj1986151942@nwafu.edu.cn (J.D.); pjq055283@nwafu.edu.cn (J.P.); ljy1007@nwafu.edu.cn (J.L.); ptl1997@nwafu.edu.cn (T.P.); 2020011877@nwafu.edu.cn (G.C.); wangrongqun@nwafu.edu.cn (R.W.); songshenh@nwafu.edu.cn (S.H.); lixiaojing@nwafu.edu.cn (X.L.); 2 Key Laboratory of Protected Horticultural Engineering in Northwest, Ministry of Agriculture, Yangling 712100, China; 3 Shaanxi Protected Agriculture Research Centre, Yangling 712100, China

**Keywords:** tomato (*Solanum lycopersicum*), DC1 domain, SlCHP, gene family analysis, abiotic stresses

## Abstract

DC1 (Divergent C1) domain proteins are a new class of proteins that have been discovered in recent years, which play an important role in plant growth, development, and stress response. In order to better study the distribution and function of DC1 domain proteins in tomatoes, a genome-wide identification was conducted. It was found that there are twenty-one DC1 domain protein genes distributed on nine chromosomes of tomatoes, named *SlCHP1-21*. Phylogenetic analysis shows that twenty-one *SlCHP* genes are divided into six subfamilies. Most of the *SlCHP* genes in tomatoes have no or very short introns. All SlCHP proteins, with the exception of SlCHP8 and SlCHP17, contain variable amounts of C1 domain. Analysis of the *SlCHP* gene promoter sequence revealed multiple cis-elements responsive to plant stress. qRT-CR analysis showed that most members of *SlCHP* gene expressed in the roots. The *SlCHP11*, *13*, *16*, *17,* and *SlCHP20* genes showed specific responses to high temperature, low temperature, salt, and drought stress. In addition, the subcellular localization and interaction proteins of SlCHP were analyzed and predicted. Together, these results provides a theoretical basis for further exploration of the function and mechanism of the *SlCHP* gene in tomatoes.

## 1. Introduction

The diversity of plant growth environments determines that plants will encounter various adverse conditions during their growth process, such as high temperature, low temperature, salinity, and drought, which will pose significant challenges to the growth, development, and reproduction of plants [1,2,3,4]. In response to these stresses, plants have evolved a range of signaling mechanisms to deliver stimuli to different compartments of the cell in response to these stresses [5,6,7]. Divergent C1 (DC1) domain proteins (CHP) are a class of atypical PKC (protein kinase C) containing proteins that may play important roles in plant growth, development, and stress response [8].

The DC1 domain is a cysteine and histidine-rich zinc-finger domain of approximately 50 amino acids, named for its structural similarity to the C1 domain in protein kinase C and other mammalian proteins [9,10]. DC1 domain proteins in plants generally contain multiple C1 domains, so they are also called Divergent C1 domain proteins in plants [11]. PKCs play an important role in cell growth, differentiation, cell metabolism, and transcriptional activation, and it has four conserved domains, namely C1, C2, C3, and C4 [12,13,14]. C1 and C2 are structurally distinct from other protein kinases in that they bind Ca^2+^, phospholipids, DAG (diacylglycerol), and PA (phosphatidic acid), and the C1 and C2 domains are also called regulatory domains. The C3 domain includes an ATP-binding sequence, Gly-X-Gly-X-X-Gly-Lys, which has high homology with the ATP-binding sites of other protein kinases, also known as the catalytic region. The C4 domain contains a substrate binding region and is required for the recognition of phosphorylated substrates [9,15,16]. The C1 domain was initially identified in PKC via phosphatidyl binding DAG and phorbol ester and was later found to bind proteins by transporting them to membranes, where they can interact with signaling complexes, substrates and regulators, substrate enzyme activation, and intermembrane transport [17,18]. However, it is still unknown whether plant DC1 domain proteins bind DAGs, which means that DC1 domain proteins in plants may have different functions from those in animals [16].

Some DC1 domain proteins have been identified in plants, which are involved in disease resistance, stress tolerance, and growth and development. In Arabidopsis, DC1 domain protein ULI3 was found to affect plant photomorphogenesis, localize in the cytoplasm, and participate in the mechanism of UV photoprotection [19]. VLG (VACUOLELESS GAMETOPHYTES) is localized to the vacuolar membrane, and its mutation prevents the formation of vacuoles during early gametophyte development, thereby affecting mitosis and causing abnormal seed development in Arabidopsis [11]. Another DC1 domain protein BINUCLEATE POLLEN (BNP) also regulates the development of Arabidopsis pollen [20]. The *NtDC1A* and *NtDC1B* genes in tobacco are induced by Streptococcus and may be involved in phenylpropane metabolism to specifically regulate the defense response of BY-2 cells [21]. A DC1 domain protein found in pepper can positively regulate plant pathogen infection. Infection with *Xanthomonospora capsicum* (Xcv) induces the expression of *CaDC1*, and silencing *CaDC1* increases pepper susceptibility to Xcv infection, accompanied by decreased salicylic acid accumulation and decreased expression of defense-related genes [22].

DC1 domain proteins also play important roles in plant responses to abiotic stresses. The DC1 domain gene *At5g17960* in Arabidopsis can be induced by some hormones and stress, which may be involved in hormone induced stress responses in plants [8]. The DC1 domain gene *TaCHP* in wheat exhibits differential expression between salt-tolerant and salt-sensitive varieties. Overexpression of *TaCHP* significantly improves the performance of wheat under salt stress. The cis-elements ABRE, MYBRS, and MYCRS are found in the upstream region of the *TaCHP* open reading frame, which indicates that it is a component of the ABA signal transduction pathway related to plant response to Abiotic stress [23,24]. *GhCHR* is a DC1 domain gene in cotton, which is highly expressed in cotton seedlings. Ectopic expression of *GhCHR* in Arabidopsis can confer salt stress tolerance by reducing Na^+^ accumulation in plants and improving primary root growth [25].

Seventy-three DC1 domain proteins have been identified in Arabidopsis [8]. However, as a valuable model species for investigating the developmental and postharvest biology of horticultural crops, as well as its economic significance as a widely cultivated fleshy fruit crop, there have been no reports on the DC1 domain proteins in tomatoes (*Solanum lycopersicum*. L). In this study, all DC1 domain genes in tomatoes were genome-wide identified, and their chromosomal distribution, gene structure, evolutionary relationship, tissue expression, and induced expression under different stress conditions were analyzed. A number of DC1 domain proteins that respond to abiotic stress were identified. Our results have established a fundamental understanding of DC1 domain genes in tomatoes, laying a foundation for conducting further investigations into their functional mechanisms.

## 2. Results

### 2.1. Identification of DC1 Domain Family Members in Tomatoes

A total of 21 DC1 domain family members were identified in the tomato genome and named *SlCHP1*~*SlCHP21* based on their distribution on chromosomes (Table 1). We analyzed the ID (SGN, https://solgenomics.net/, accessed on 15 march 2023), amino acid (aa) length, isoelectric point (pI), molecular weight (MW) of all *SlCHP* genes, and predicted the subcellular localization of 21 SlCHP proteins. *SlCHP* cDNA sequence length between 336~1602 bp, the length is between 111~533 amino acids, the molecular weight is between 12.736~61.624 kD. Among them, the *SlCHP18* gene had the largest molecular weight, cDNA length was 1602 bp, its length was 533 amino acid residues, and its molecular weight was 61.624 kD. SlCHP8 has a cDNA length of 336 bp, its length was 111 amino acid residues, and a molecular weight of 12.736 kD, which is the smallest among all the genes. The isoelectric point (pI) of the *SlCHP* protein varied from 4.67 to 9.38, with *SlCHP15* having the highest pI of 9.38. The lowest is *SlCHP17*, with a pI of 4.67. The subcellular localization prediction results showed that the 21 family members were mainly localized in the cytoplasm, nucleus, extracellular, chloroplast, and peroxisome. Among them, 11 members (SlCHP1, 2, 3, 4, 5, 7, 11, 13, 15, 16, 21) are located in the nucleus, 4 members (SlCHP8, 14, 17, 18) are located in the chloroplast, 3 members (SlCHP10, 19, 20) are located in the cytoplasm, and SlCHP6 may be located in the peroxisome, *SlCHP9* may be located outside the cell, and *SlCHP12* may be located in both the cytoplasm and nucleus.

### 2.2. Chromosomal Distribution, Gene Structure, and Protein Conservation Domain Analysis of SlCHP Genes

**Figure 1:**
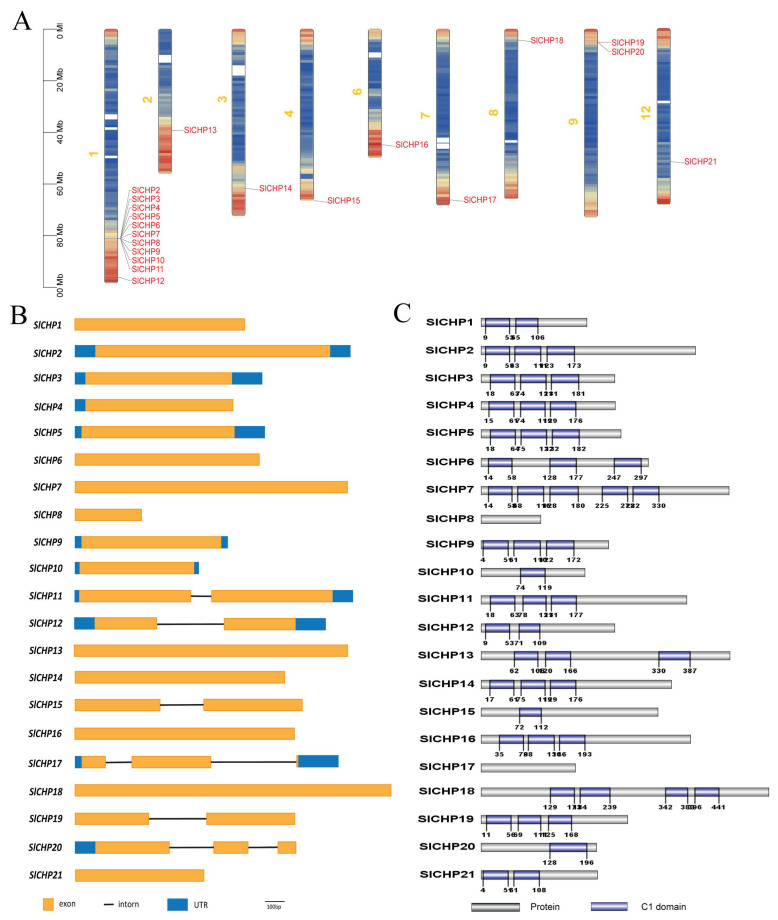
The chromosomal distribution, gene structure, and protein conservation domain analysis of C1 domain gene. (**A**) The distribution of DC1 domain genes on chromosomes. Different colors indicate the density of genes on chromosomes, red for higher gene density, and blue for lower gene density. (**B**) The exon and intron structures of tomato *SlCHP* genes. (**C**) Conserved domain analysis of tomato SlCHP proteins.

We found that 21 *SlCHP* genes were distributed on 9 chromosomes. There are 12 on chromosome 1, 2 on chromosome 9, and one on chromosomes 2, 3, 4, 6, 7, 8, and 122, respectively. Interestingly, 10 out of the 11 *SlCHP* genes on chromosome 1 exist in the form of gene clusters. *SlCHP1* (SGN: https://solgenomics.net/locus/8700/view, accessed on 15 March 2023) is not displayed on chromosome 0 (Figure 1A). Next, we analyzed the gene structure of *SlCHP* genes and found that most *SlCHP* genes do not have introns. Among the 21 *SlCHP* genes, 15 do not have introns. The remaining *SlCHP11*, *SlCHP12*, *SlCHP15*, *SlCHP19* genes contain one intron, *SlCHP17* and *SlCHP20* genes contain two introns, and these introns are all short (Figure 1B).

We analyzed the conserved domains of SlCHP proteins using the NCBI website database (https://www.ncbi.nlm.nih.gov/cdd, accessed on 15 March 2023) and found that all SlCHP proteins contained one or more C1 domains, except SlCHP8 and SlCHP17, which did not have C1 domains and may be pseudogenes. Among them, SlCHP10, SlCHP15, and SlCHP20 contain 1 C1 domain, SlCHP1, SlCHP12, and SlCHP21 contain 2 C1 domains, SlCHP18 contains 4 C1 domains, SlCHP7 contains 5 C1 domains, and the remaining SlCHP proteins all contain 3 C1 domains (Figure 1C). These results indicate that the SlCHP protein in tomatoes also exists in the form of divergent C1 domains, which is consistent with the previous belief that CHP proteins in plants generally contain divergent C1 domains [11].

### 2.3. Phylogenetic Analysis of the SlCHP Genes in Tomatoes

**Figure 2:**
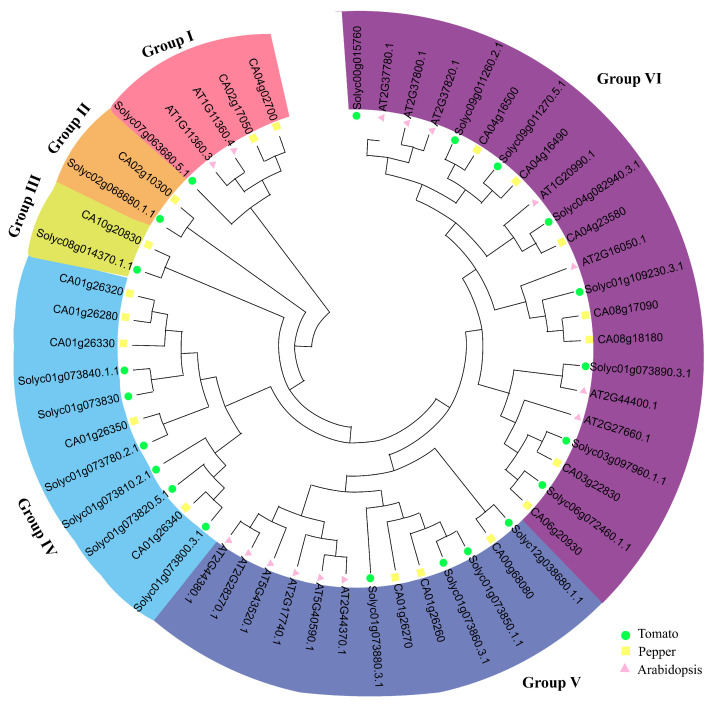
Phylogenetic analysis of the tomato *SlCHP* genes. Different colors indicate different subgroups.

We conducted a phylogenetic tree analysis of *CHP* genes in tomato, Arabidopsis and peppers, which showed that the 21 *SlCHP* genes of tomatoes were divided into 6 subgroups (Figure 2). *SlCHP17*(*Solyc07g063680.5.1*) was a separate subgroup; *SlCHP13* (*Solyc02g068680.1.1*) was a subgroup; *SlCHP18* (*Solyc08g014370.1.1*) was a subgroup; *SlCHP2* (*Solyc01g073780.2.1*), *SlCHP3* (*Solyc01g073800.3.1*), *SlCHP4* (*Solyc01g073810.2.1*), *SlCHP5* (*Solyc01g073820.5.1*), *SlCHP6* (*Solyc01g073830.1*), and *SlCHP7* (*Solyc01g073840.1.1*) was a subgroup and have close genetic relationships and are also gene clusters on chromosome 1, which may exercise the same type of function in the form of gene clusters; *SlCHP8* (*Solyc01g073850.1.1*), *SlCHP9* (*Solyc01g073860.3.1*), *SlCHP10* (*Solyc01g073880.3.1*), and *SlCHP21*(*Solyc12g038680.1.1*) was a subgroup; the remaining *SlCHP1*, *SlCHP11*, *SlCHP12*, *SlCHP14*, *SlCHP15*, *SlCHP16*, *SlCHP19*, and *SlCHP20* were on a large clade and divided into another subgroup.

### 2.4. Analysis of Cis-Elements in the Promoter of the SlCHP Gene Family

**Figure 3:**
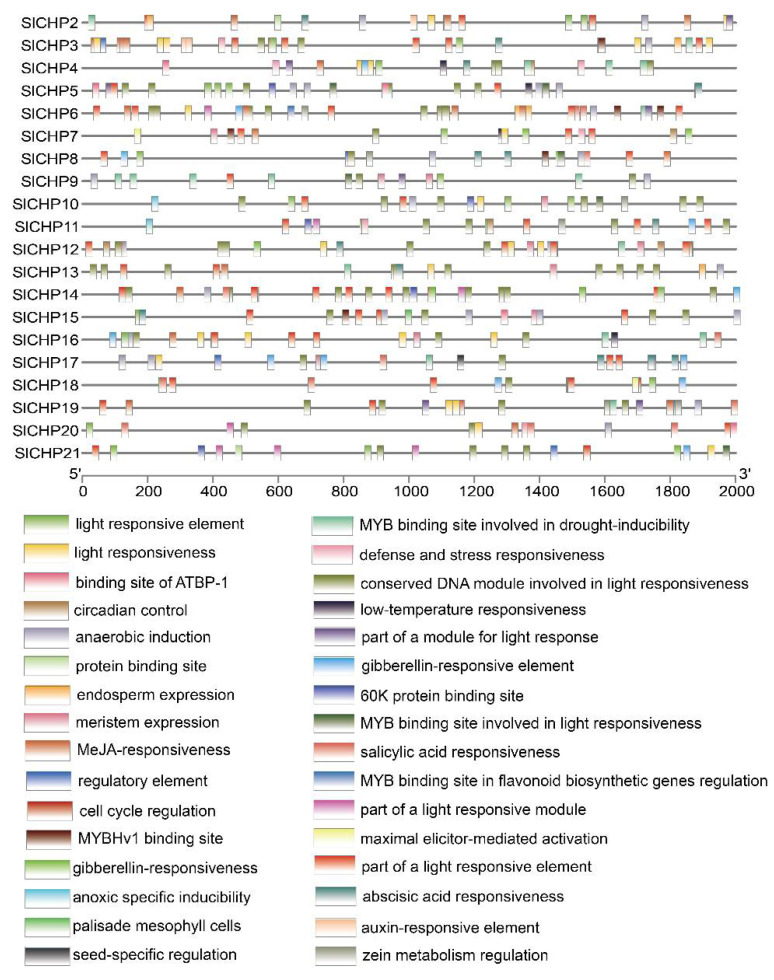
Analysis of cis-acting elements of tomato *SlCHP* gene promoters. This line represents the upstream 2000 bp promoter of the *SlCHP* genes, with different colored rectangles representing different cis-elements.

We used the Plant CARE database (http://bioinformatics.psb.ugent.be/webt-ools/plantcare/html/, accessed on 12 April 2023) to predict and analyze cis-acting elements in 2000 bp upstream of the start codon of the *SlCHP* genes promoter region. The results showed that CAAT-box (a common cis-acting element in promoter and enhancer regions) and TATA-box (a core promoter element for transcription initiation) were highly contained in the promoter regions of 21 tomato *SlCHP* gene family members. There are also many stress response-related elements, such as a G-box element related to light response, an LTR element related to low temperature response, a WUN-motif element related to trauma response, an MBS element related to drought stress, and a TC-rich element related to defense and stress response. In addition, there are various hormone responsive cis-acting elements, such as ethylene responsive element ERE, salicylic acid responsive element TCA-element, abscisic acid responsive element ABRE, gibberellin responsive element GARE-motif, methyl jasmonate responsive element CGTCA-motif and TGACG-motif, and plant hormone responsive element TGA-box (Figure 3). This indicates that the tomato *SlCHP* gene family is closely related to abiotic stress response and growth and development.

### 2.5. Analysis of SlCHP Family Genes Tissue Expression

**Figure 4:**
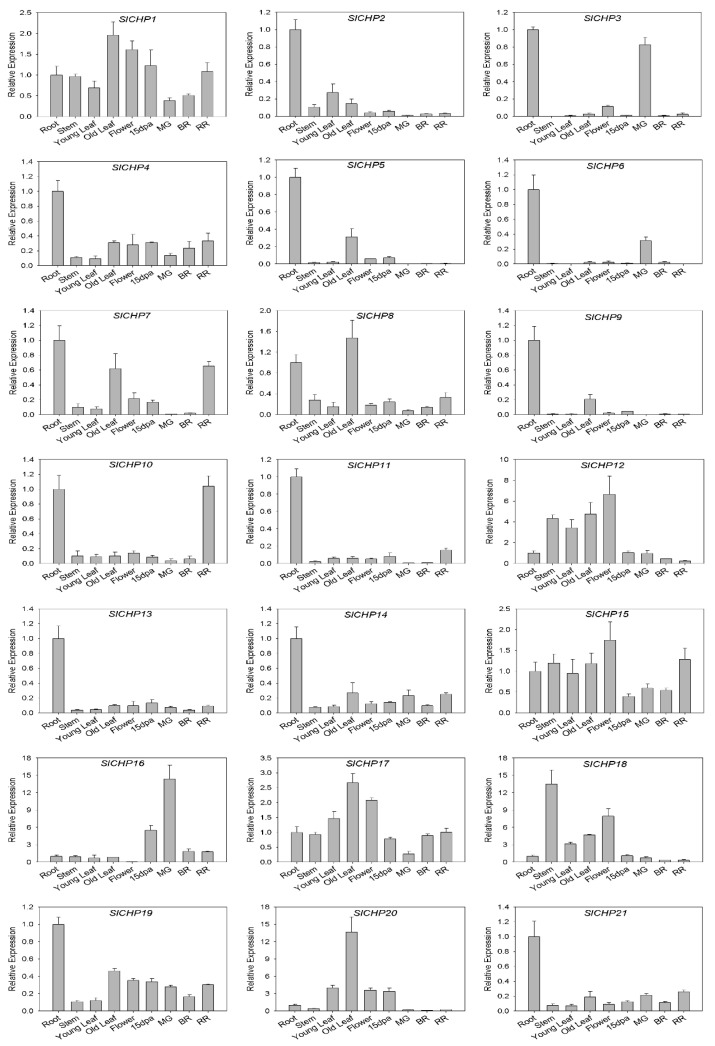
Expression profiles of the tomato *SlCHP* genes. dpa: day post-anthesis; MG: mature green; BR: breaker; RR: red ripen.

In order to further understand the expression of SlCHP genes in tomatoes, we detected the tissue expression profiles of 21 *SlCHP* genes in tomato tissues by RT-qPCR (Figure 4). It is obvious that the expression pattern of the *SlCHP2*-*SlCHP11* gene cluster is very similar, mainly expressed in the root, while the expression level is very low in other tissues. Similarly, *SlCHP13*, *SlCHP14*, *SlCHP19*, and *SlCHP21* are mainly expressed in the root, with low expression levels in other tissues. It indicates that most genes in the *SlCHP* family are likely to primarily function in the root and are closely related to root development or nutrient absorption. In addition, the expression level of *SlCHP18* in stems was higher, indicating that it might be related to stem development, and the expression level of *SlCHP20* in old leaves was higher, which might be related to leaf senescence. The expression level of *SlCHP16* is high in fruits and low in flowers, suggesting that *SlCHP16* may play a positive regulatory role in fruits and a negative regulatory role in flowers.

### 2.6. Expression Analysis of SlCHP Genes under Several Abiotic Stresses

**Figure 5:**
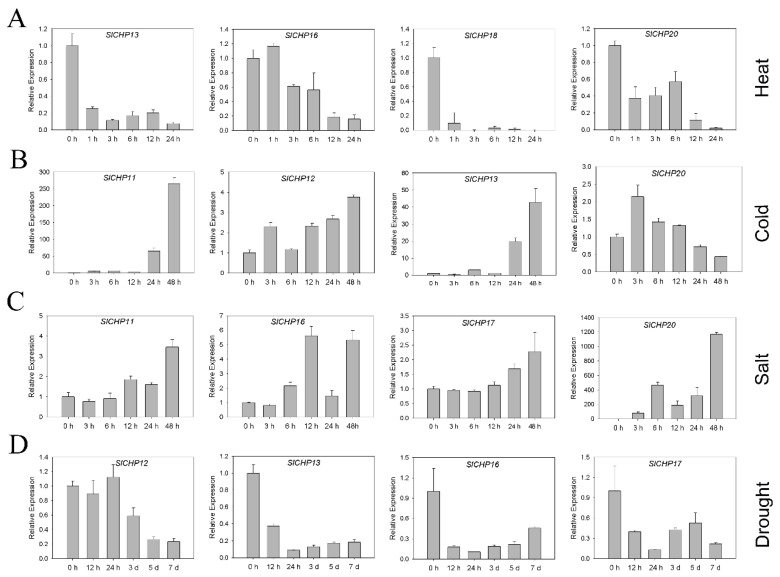
Induced expression of *SlCHP* genes under different stresses. (**A**) Induced expression of *SlCHP* genes under heat stress; (**B**) cold stress; (**C**) salt stress; (**D**) drought stress.

We analyzed the induced expression of *SlCHP* genes under heat, cold, salt, and drought abiotic stress. It was found that *SlCHP13*, *SlCHP16*, *SlCHP18,* and *SlCHP20* responded to heat stress and were inhibited, and the expression levels of *SlCHP13* and *SlCHP18* were significantly decreased within 1 h of high temperature, while the expression levels of *SlCHP16* and *SlCHP20* were significantly decreased within 6 h of high temperature (Figure 5A). Under cold stress, the expressions of *SlCHP11*, *SlCHP12*, and *SlCHP13* were upregulated; *SlCHP11* and *SlCHP13* were particularly strongly induced after low temperature 24 h, while *SlCHP20* was inhibited by cold stress (Figure 5B). *SlCHP11*, *SlCHP16*, *SlCHP17*, and *SlCHP20* were induced under salt stress; *SlCHP20* was especially induced by salt stress (Figure 5C). *SlCHP12*, *SlCHP13*, *SlCHP16*, and *SlCHP17* were inhibited under drought stress (Figure 5D). It is suggested that *SlCHP* genes may play a role in abiotic stress response and resistance formation.

### 2.7. Subcellular Localization of the SlCHP Proteins

**Figure 6:**
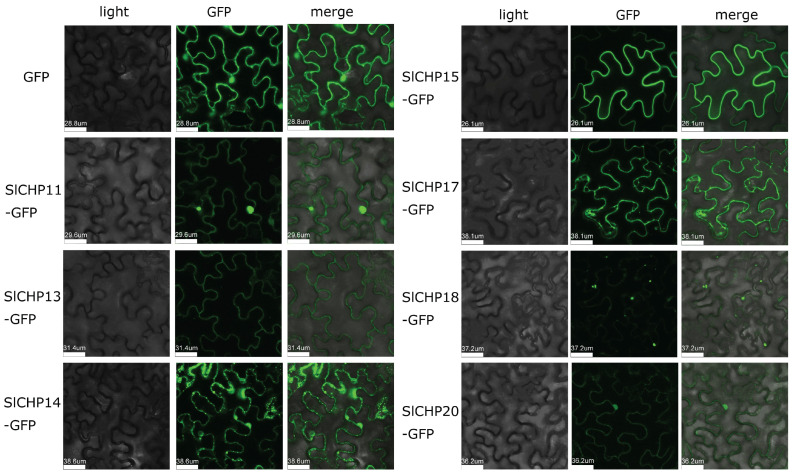
Subcellular localization of SlCHP-green fluorescent protein (GFP) fusion proteins.

In order to further understand the function of tomato SlCHP proteins, subcellular localization of a part of SlCHP protein was performed. The SlCHP protein fused with GFP protein was instantaneously expressed in tobacco leaves under the drive of CaMV35S promoter. Observing fluorescence signals through confocal microscopy showed that SlCHP11 was located in the nucleus and cytoplasm, SlCHP13 and SlCHP15 were located in the cell membrane, SlCHP17 was located in the cytoplasm, SlCHP20 was located in the nucleus and membrane, and SlCHP14 and SlCHP18 were located in unclear organelles (Figure 6). These data provide some insights for understanding the C1 domain protein.

### 2.8. Prediction Interaction Protein Prediction of SlCHP Proteins

**Figure 7:**
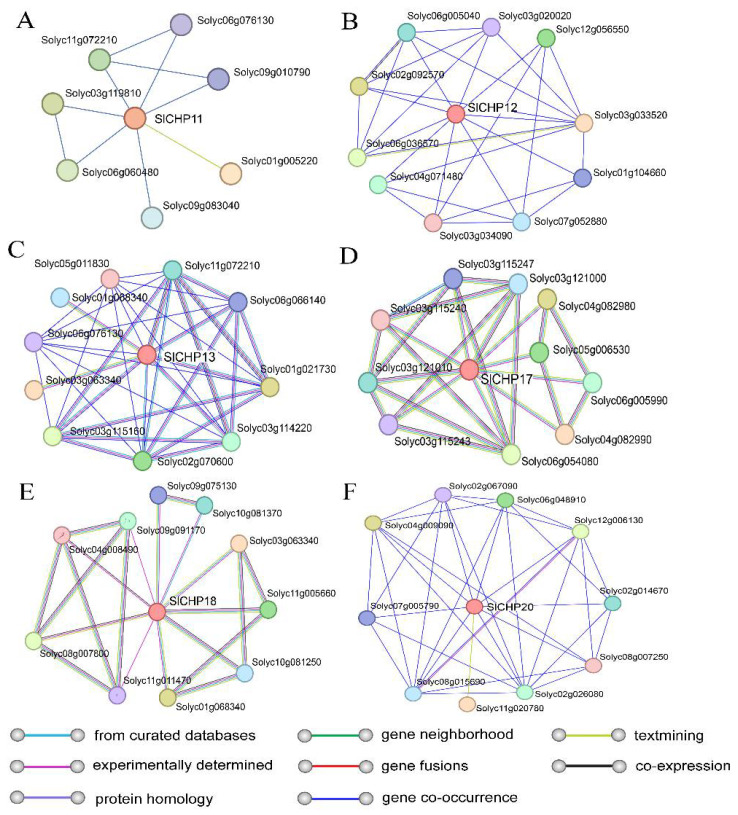
Predicted protein association networks analyses of SlCHP proteins: (**A**) SlCHP11; (**B**) SlCHP12; (**C**) SlCHP13; (**D**) SlCHP17; (**E**) SlCHP18; (**F**) SlCHP20. The nodes represent the proteins, and the different color lines represent the protein–protein associations.

To further understand the possible regulatory pathways of SlCHP proteins, we used the STRING database (https://string-db.org/cgi/, accessed on 12 April 2023) to predict the protein–protein interaction relationships of SlCHP proteins (Figure 7). The interaction proteins associated with SlCHP11, SlCHP12, and SlCHP20 are relatively few, and are mainly predicted through gene co-occurrence pathways (Figure 7A,B,F). SlCHP13, SlCHP17, and SlCHP18 have more proteins related to each other, including from curated databases, experimentally determined gene neighborhoods, and co-expression pathway (Figure 7C–E). And there is less overlap between the predicted interaction proteins of different SlCHP proteins, suggesting that C1 domain proteins may play a variety of functions in tomatoes.

## 3. Discussion

The DC1 domain in plants is a type of zinc-finger domain rich in cysteine and histidine [9,20]. The biggest feature different from animals is that there is no separate C1 domain in plants, but rather a protein composed of a Divergent C1 domain containing multiple C1 domains [11]. There are few studies on the function and mechanism of DC1 domain proteins in plants, and little research has been reported in tobacco, pepper, Arabidopsis, wheat, and cotton [11,20,21,22,23,25], while there are no reports of DC1 domain proteins in tomatoes. To systematically understand the DC1 domain protein in tomatoes and explore new stress resistance regulatory factors, this study conducted a genome-wide analysis of the DC1 domain protein gene in tomatoes. A total of 21 *SlCHP* genes were identified, and there were fewer DC1 domain protein genes in tomatoes compared with the 73 DC1 domain genes in Arabidopsis [8]. Meanwhile, the Phylogenetic of 21 *SlCHP* genes in tomatoes was divided into 6 subfamilies, which might be related to gene functional differentiation.

Gene duplications including segmental and tandem duplications and inversion events are universal dynamics leading to expansion of family members and genome complexity in eukaryotes [26,27]. The duplication of two genes located on one chromosome is a tandem duplication event, whereas gene duplications occurring on different chromosomes are identified as segmental duplications [28,29]. The *SlCHP* genes are distributed on nine chromosomes, except for *SlCHP1*, which is not assembled on a defined chromosome and is labeled chromosome 0. Half of the *SlCHP* genes (*SlCHP2*-*11*) are located on chromosome 1 and exist in the form of gene clusters. These genes may have been originally produced by tandem replication of the same gene, and they may exercise the same type of function (Figure 1A). In the process of plant evolution, most genes have long and multiple introns [30]; however, most *SlCHP* genes do not have introns, 15 out of 21 *SlCHP* family genes do not have introns, four *SlCHP* genes have one intron, two *SlCHP* genes have two introns, and the introns are all very short (Figure 1B). This is an interesting phenomenon, as the *SlCHP* family genes in tomatoes may have been replicated over a relatively recent period of time. With the exception of SlCHP8 and SlCHP17, which have no C1 domain and may be pseudogenes, all other SlCHP proteins contain one or more C1 domains, indicating that the SlCHP protein in tomatoes conforms to the characteristic domain of typical plant DC1 domain proteins. By analyzing the gene structure and conserved motifs of the DC1 domain family in tomatoes, more clues can be provided for the evolutionary relationship of plant DC1 domain genes.

The only known functions of DC1 domain genes are involved in plant growth and development and biotic and abiotic resistance, indicating the diversity of DC1 domain proteins. Due to the fact that gene expression can provide important clues to gene function [31,32], in order to understand the function of DC1 domain protein in tomatoes, we analyzed the promoter and expression of DC1 domain protein genes in tomatoes. Promoter analysis revealed a large number of cis-elements related to hormone and abiotic stress responses on the promoters of the *SlCHP* family genes (Figure 3), suggesting that the *SlCHP* gene may be more involved in abiotic stress in tomatoes. Tissue expression analysis revealed that most of the *SlCHP* genes were highly expressed in the root. The root of plants determine their ability to acquire and distribute nutrients and water [33,34], suggesting the possibility of *SlCHP* genes improving tomato environmental adaptability. The similarity of such a large number of expression patterns also indicates the conservatism of tomato *SlCHP* gene evolution. Through the induction treatment of several abiotic stresses such as high temperature, low temperature, salt, and drought, we found that several genes such as *SlCHP11*, *SlCHP13*, *SlCHP16*, and *SlCHP20* were induced by various abiotic stresses. However, genetic evidence is essential to determine whether a gene is related to a certain biological function. Next, the roles of these genes in abiotic stress in tomatoes can be further determined by transgenic techniques to provide genetic resources for improving resistance traits of tomato varieties.

Subcellular localization showed that 7 SlCHP proteins were localized to various organelles, which is consistent with previous predictions (Table 1, Figure 6), including the nucleus, cell membrane, cytoplasm, and other organelles. This indicates that although the SlCHP protein belongs to the DC1 domain protein family, their functions and mechanisms may be different. The prediction of the interaction proteins of some SlCHP proteins showed that the interaction network of some SlCHP proteins was relatively simple, such as SlCHP11, while the interaction proteins of other SlCHP proteins were more complex, such as SlCHP13 (Figure 7). These interactions suggest that SlCHP proteins may play important roles in forming complexes with other proteins during tomato growth and development and in response to various stresses. These interaction information provided certain reference information for revealing the mechanism of SlCHP protein in the future.

In summary, this study conducted genome-wide identification of all DC1 domain genes in tomatoes. There were a total of 21 *SlCHP* genes distributed on nine chromosomes, and phylogenetic analysis showed that they were divided into six subgroups. Tissue expression and stress-induced expression of all *SlCHP* genes showed that most *SlCHP* genes were highly expressed in root, and some *SlCHP* genes were induced by multiple stresses. At the same time, we identified the key cis-elements of the *SlCHP* gene promoter region and conducted protein–protein interaction and gene co-expression analysis to further evaluate the function of SlCHP in tomato growth, development, and stress response. The identification and characterization of the *SlCHP* gene family in tomatoes laid a good foundation for genetic improvement and provided theoretical basis and guidance for further research on the functions of plant DC1 domain proteins.

## 4. Materials and Methods

### 4.1. Sequence Retrieval and Identification of Tomato DC1 Domain Family Members

The genome sequence, protein sequence, and genome annotation files of tomatoes were downloaded from the National Center for Biotechnology Information (NCBI, https://www.ncbi.nlm.nih.gov/, accessed on 15 March 2023) database, while the sequence information of DC1 domain proteins was obtained from the Sol Genomics Network (SGN, https://solgenomics.net/, accessed on 15 March 2023) website [35,36]. Using the protein sequence of the DC1 domain protein gene family from Arabidopsis (TAIR, http://www.arabidopsis.org/, accessed on 15 March 2023) as the alignment sequence, a BLAST database was locally constructed to preliminarily screen for predicted members of the tomato DC1 domain protein gene family. The conserved C1 domain from SlCHP16 were used as a seed sequence in NCBI (AEKE00000000.3) and SGN (SL4.0) databases for sequence search through BLASTP to identify all SlCHP genes. Next, the HMMER [37] procedures of hmm build and hmm search were fully used to retrieve all assumed DC1 domain sequences (Pfam36.0: PF03107) with default parameters in tomatoes, and collect the ID of the relevant sequences to ensure that all DC1 domain genes are retrieved (E-value < 1.0) [38].

### 4.2. Physical and Chemical Properties Analysis of Tomato DC1 Domain Protein

The ProtParam tool on the online website ExPAsy (http://www.expasy.org/, accessed on 12 April 2023) is used to analyze the physicochemical properties of various members of the tomato DC1 domain protein genome [39]. Input the amino acid sequence of DC1 domain protein to obtain the number of amino acids, molecular weight, and isoelectric point (pI), and predict the subcellular localization of DC1 domain protein.

### 4.3. Chromosomal Location, Gene Structure, and Conservative Domain Analysis

The exon and intron distribution structure of DC1 domain gene was obtained from the SGN database (https://solgenomics.net/search/locus, accessed on 12 April 2023), and the conserved domain information of DC1 domain protein was obtained from the protein CDD database (https://www.ncbi.nlm.nih.gov/cdd, accessed on 12 April 2023) of NCBI. Using the gene location function in TBtools v1.108 software (Guangzhou, China) [40], based on the GFF annotation file and gene names, plot the distribution location information of all DC1 domain genes on chromosomes and obtain a display map [41].

### 4.4. Phylogenetic Analysis

Multiple sequence alignments were performed on DC1 domain genes in tomatoes, Arabidopsis, and peppers (*Capsicum annuum*) using ClusterX1.81 [42]. The phylogenetic tree of DC1 domain protein was constructed by MEGA 6.0 software. The evolutionary history was inferred by neighbor–neighbor method (NJ), and the Bootstrap was set to 1000 replicates [43].

### 4.5. Promoter Analysis

A 2000 bp promoter sequence of the DC1 domain gene start codon upstream was downloaded from the SGN database (https://solgenomics.net/, accessed on 12 April 2023) and submit to Plant CARE [44] (http://bioinformatics.psb.ugent.be/webt-ools/plantcare/html/, accessed on 12 April 2023) and Plant PAN4.0 [45] (http://plantpan.itps.ncku.edu.tw/plantpan4/index.html/, accessed on 12 April 2023) online websites to analyze the cis-elements and predict their functions. The identified CAREs were visualized using the Toolkit Biologists (TBtools) integrated with various biological data processing tools [40].

### 4.6. Protein Interaction Network Prediction

STRING database (https://string-db.org/cgi/, accessed on 12 April 2023) [46] was used to predict the protein–protein in-teraction relationships same of SlCHP proteins. The data output from the STRING database was visualized in Cytoscape software 3.6.1 [47].

### 4.7. Plant Material and Growth Condition

A *Solanum lycopersicum* cultivar Alisa Craig (AC) was used in this study. Seeds were seeded in 50-well plates filled with substrate containing peat, vermiculite and perlite, and tomato seedlings were cultivated in climate chambers with normal conditions (25 °C/18 °C and 12 h day/12 h night), relative humidity of 60% and photosynthetic photon flux density of 300 µmol·m^−2^·s^−1^. On the 30th day after sowing, the seedings were individually transplanted into a plastic pot (5 cm × 5 cm × 8 cm) and continued to grow for 10 days. Six-leaf tomato seedlings with a similar growth were chosen for the abiotic stress treatment to check the expression of genes. Low-temperature treatment: 4 °C/4 °C and 12 h day/12 h night. High-temperature treatment: 42 °C/42 °C and 12 h day/12 h night. Salt treatment: add 200 mM NaCl solution to the substrate (100 mL/plant). Drought stress: Fill the tray with water to soak the substrate. When the substrate no longer absorbs water, pour out the water in the tray and do not water it again later. The second functional leaf of tomato were taken at 0, 3, 6, 12, 24, and 48 h for high temperature stress, at 0, 3, 6, 12, 24, 48, and 96 h for low temperature and salt stress, and at 0 h, 12 h, 24 h, 3 d, 5 d, and 7 d for drought stress. The samples is frozen in liquid nitrogen and stored in −80 °C, with three biological replicates at each time point.

### 4.8. RNA Extraction and Real-Time Quantitative PCR Assay

Total RNA from frozen tissue was extracted using TRIzol reagent. Then, 5 µg RNA were reverse transcribed into complementary DNA (cDNA) using HiScript II 1st Strand cDNA Synthesis Kit (Vazyme, Nanjing, China). Real-time quantitative PCR (RT-qPCR) based on SYBR green was used to detect gene transcription level using cDNA as template. The RT-qPCR was conducted using the Applied Biosystems VIIA6 Fast Real-Time PCR System according to the manufacturer’s instructions. Three independent biological replicates were carried out for all assays. The tomato actin gene was used as a reference gene, and its relative expression levels were calculated using the 2^−∆∆Cт^ method [48,49]. The primer sequences for reference genes and related genes are shown in Supplementary Table S1.

### 4.9. Subcellular Localization

For subcellular localization analysis, the full-length coding sequence (CDS) without the stop codon of the DC1 domain gene was used to construct the GFP2300 vector to form a vector fused with green fluorescent protein (GFP). The final vector was transferred into *Agrobacterium tumefaciens* GV3101. Four-week-old tobacco (*Nicotiana benthamiana*) leaves were infiltrated with this *A. tumeticum* using the method previously described and cultured for 48 h in low light [50]. The tobacco leaves were collected and fluorescence from GFP was visualized using a confocal laser scanning microscope (Leica TCS SP8, Germany). Subcellular localization experiment completed at the Horticulture Science Research Center at College of Horticulture, NWAFU.

## Figures and Tables

**Table 1:** *SlCHP* gene family member information.

No.	Gene Name	Gene ID	Chromosome Location	cDNA	AA	pI	MW (kDa)	Locali-Zation
1	*SlCHP1*	Solyc00g015760.1.1	Ch00: 11237737-11236877 (−)	861	286	6.89	31.521	Nucl
2	*SlCHP2*	Solyc01g073780.2.1	Ch01: 81154238-81152883 (−)	1194	397	5.69	45.173	Nucl
3	*SlCHP3*	Solyc01g073800.3.1	Ch01: 81158393-81157477 (−)	747	248	6.58	27.623	Nucl
4	*SlCHP4*	Solyc01g073810.2.1	Ch01: 81170807-81170011 (−)	750	249	5.96	27.624	Nucl
5	*SlCHP5*	Solyc01g073820.5.1	Ch01: 81173167-81172221 (−)	780	259	6.65	29.144	Nucl
6	*SlCHP6*	Solyc01g073830.1	Ch01: 81180355-81179423 (−)	933	310	4.82	35.355	Pero
7	*SlCHP7*	Solyc01g073840.1.1	Ch01: 81183692-81182313 (−)	1380	459	5.69	52.643	Nucl
8	*SlCHP8*	Solyc01g073850.1.1	Ch01: 81185584-81185249 (−)	336	111	5.55	12.736	Chlo
9	*SlCHP9*	Solyc01g073860.3.1	Ch01: 81194192-81193425 (−)	711	236	5.51	27.207	Extr
10	*SlCHP10*	Solyc01g073880.3.1	Ch01: 81211961-81211353 (−)	582	193	5.32	21.907	Cyto
11	*SlCHP11*	Solyc01g073890.3.1	Ch01: 81215509-81217001 (+)	1146	381	7.07	42.222	Nucl
12	*SlCHP12*	Solyc01g109230.3.1	Ch01: 96240517-96242373 (+)	747	248	9.03	28.467	Nucl or Cyto
13	*SlCHP13*	Solyc02g068680.1.1	Ch02: 38624575-38625960 (+)	1386	461	7.31	53.235	Nucl
14	*SlCHP14*	Solyc03g097960.1.1	Ch03: 60337788-60336727 (−)	1062	353	7.88	38.769	Chlo
15	*SlCHP15*	Solyc04g082940.3.1	Ch04: 66412733-66411463 (−)	930	328	9.38	35.681	Nucl
16	*SlCHP16*	Solyc06g072460.1.1	Ch06:44695610-44696722 (+)	1167	370	8.29	40.426	Nucl
17	*SlCHP17*	Solyc07g063680.5.1	Ch07: 66082531-66086025 (+)	528	175	4.67	19.024	Chlo
18	*SlCHP18*	Solyc08g014370.1.1	Ch08: 4293649-4295250 (+)	1602	533	7.26	61.624	Chlo
19	*SlCHP19*	Solyc09g011260.2.1	Ch09: 4592852-4594526 (+)	819	272	8.27	31.079	Cyto
20	*SlCHP20*	Solyc09g011270.4.1	Ch09: 4603003-4605126 (+)	645	214	8.35	24.910	Cyto
21	*SlCHP21*	Solyc12g038680.1.1	Ch12: 47165974-47165324 (−)	651	216	8.80	24.740	Nucl

AA: number of amino acids; pI: theoretical isoelectric point; MW: molecular weight (kDa); Cyto: cytoplasm; Extr: extracellular; Nucl: nucleus; Chlo: chloroplast; Pero: peroxisome.

## Data Availability

Data are contained within the article and Supplementary Materials.

## Supplementary Materials

The following supporting information can be downloaded at: https://www.mdpi.com/article/10.3390/ijms242316994/s1.

## References

[1] Willis C.G., Ruhfel B., Primack R.B., Miller-Rushing A.J., Davis C.C. (2008). Phylogenetic patterns of species loss in Thoreau’s woods are driven by climate change. Proc. Natl. Acad. Sci. USA.

[2] Li G., Peng T., Qu F., Wang J., Long Y., Hu X. (2023). Bacillus methylotrophicus Could Improve the Tolerance and Recovery Ability of the Tomato to Low-Temperature Stress and Improve Fruit Quality. Agronomy.

[3] Cao Y., Song H., Zhang L. (2022). New Insight into Plant Saline-Alkali Tolerance Mechanisms and Application to Breeding. Int. J. Mol. Sci..

[4] Ding Y., Shi Y., Yang S. (2020). Molecular Regulation of Plant Responses to Environmental Temperatures. Mol. Plant.

[5] Qiu J., Ni L., Xia X., Chen S., Zhang Y., Lang M., Li M., Liu B., Pan Y., Li J. (2022). Genome-Wide Analysis of the Protein Phosphatase 2C Genes in Tomato. Genes.

[6] Li B., Gao K., Ren H., Tang W. (2018). Molecular mechanisms governing plant responses to high temperatures. J. Integr. Plant Biol..

[7] Ding Y., Shi Y., Yang S. (2019). Advances and challenges in uncovering cold tolerance regulatory mechanisms in plants. New Phytol..

[8] Bhaskar R.V., Mohanty B., Verma V., Wijaya E., Kumar P.P. (2015). A hormone-responsive C1-domain-containing protein At5g17960 mediates stress response in Arabidopsis thaliana. PLoS ONE.

[9] Brose N., Betz A., Wegmeyer H. (2004). Divergent and convergent signaling by the diacylglycerol second messenger pathway in mammals. Curr. Opin. Neurobiol..

[10] Gomez-Fernandez J.C., Torrecillas A., Corbalan-Garcia S. (2004). Diacylglycerols as activators of protein kinase C. Mol. Membr. Biol..

[11] D’Ippolito S., Arias L.A., Casalongue C.A., Pagnussat G.C., Fiol D.F. (2017). The DC1-domain protein VACUOLELESS GAMETOPHYTES is essential for development of female and male gametophytes in Arabidopsis. Plant J..

[12] Colon-Gonzalez F., Kazanietz M.G. (2006). C1 domains exposed: From diacylglycerol binding to protein-protein interactions. Biochim. Biophys. Acta.

[13] Quest A.F.G., Bloomenthal J., Bardes E.S.G., Bell R.M. (1992). The regulatory domain of protein kinase C coordinates four atoms of zinc. J. Biol. Chem..

[14] Buchner K. (1995). Protein Kinase C in the Transduction of Signals Toward and within the Cell Nucleus. Eur. J. Biochem..

[15] Ono Y., Fujii T., Igarashi K., Kuno T., Tanaka C., Kikkawa U., Nishizuka Y. (1989). Phorbol ester binding to protein kinase C requires a cysteine-rich__zinc-finger-like sequence. Proc. Natl. Acad. Sci. USA.

[16] Djafi N., Vergnolle C., Cantrel C., Wietrzynski W., Delage E., Cochet F., Puyaubert J., Soubigou-Taconnat L., Gey D., Collin S. (2013). The Arabidopsis DREB2 genetic pathway is constitutively repressed by basal phosphoinositide-dependent phospholipase C coupled to diacylglycerol kinase. Front. Plant Sci..

[17] Johnson J.E., Goulding R.E., Ding Z., Partovi A., Anthony K.V., Beaulieu N., Tazmini G., Cornell R.B., Kay R.J. (2007). Differential membrane binding and diacylglycerol recognition by C1 domains of RasGRPs. Biochem. J..

[18] Canagarajah B., Leskow F.C., Ho J.Y., Mischak H., Saidi L.F., Kazanietz M.G., Hurley J.H. (2004). Structural mechanism for lipid activation of the Rac-specific GAP, beta2-chimaerin. Cell.

[19] Suesslin C., Frohnmeyer H. (2003). An Arabidopsis mutant defective in UV-B light-mediated responses. Plant J..

[20] Brownfield L. (2022). Pollen Helps Reveal a Role for DC1 Domain Proteins. Plant Cell Physiol..

[21] Shinya T., Galis I., Narisawa T., Sasaki M., Fukuda H., Matsuoka H., Saito M., Matsuoka K. (2007). Comprehensive analysis of glucan elicitor-regulated gene expression in tobacco BY-2 cells reveals a novel MYB transcription factor involved in the regulation of phenylpropanoid metabolism. Plant Cell Physiol..

[22] Hwang I.S., Choi D.S., Kim N.H., Kim D.S., Hwang B.K. (2014). The pepper cysteine/histidine-rich DC1 domain protein CaDC1 binds both RNA and DNA and is required for plant cell death and defense response. New Phytol..

[23] Li C., Lv J., Zhao X., Ai X., Zhu X., Wang M., Zhao S., Xia G. (2010). TaCHP: A wheat zinc finger protein gene down-regulated by abscisic acid and salinity stress plays a positive role in stress tolerance. Plant Physiol..

[24] Tanaka N., Itoh J., Nagato Y. (2012). Role of rice PPS in late vegetative and reproductive growth. Plant Signal Behav..

[25] Gao S., Yang L., Zeng H.Q., Zhou Z.S., Yang Z.M., Li H., Sun D., Xie F., Zhang B. (2016). A cotton miRNA is involved in regulation of plant response to salt stress. Sci. Rep..

[26] Hughes A.L. (1994). The evolution of functionally novel proteins after gene duplication. Proc. Biol. Sci..

[27] Pich i Roselló O., Kondrashov F.A. (2014). Long-Term Asymmetrical Acceleration of Protein Evolution after Gene Duplication. Genome Biol. Evol..

[28] Xing H., Pudake R.N., Guo G., Xing G., Hu Z., Zhang Y., Sun Q., Ni Z. (2011). Genome-wide identification and expression profiling of auxin response factor (ARF) gene family in maize. BMC Genom..

[29] Wang R., Ming M., Li J., Shi D., Qiao X., Li L., Zhang S., Wu J. (2017). Genome-wide identification of theMADS-boxtranscription factor family in pear (Pyrus bretschneideri) reveals evolution and functional divergence. PeerJ.

[30] Schaper E., Anisimova M. (2014). The evolution and function of protein tandem repeats in plants. New Phytol..

[31] Liu M., Gomes B.L., Mila I., Purgatto E., Peres L.E., Frasse P., Maza E., Zouine M., Roustan J.P., Bouzayen M. (2016). Comprehensive Profiling of Ethylene Response Factor Expression Identifies Ripening-Associated ERF Genes and Their Link to Key Regulators of Fruit Ripening in Tomato. Plant Physiol..

[32] Huang S., Gao Y., Liu J., Peng X., Niu X., Fei Z., Cao S., Liu Y. (2012). Genome-wide analysis of WRKY transcription factors in Solanum lycopersicum. Mol. Genet. Genom..

[33] McCormack M.L., Dickie I.A., Eissenstat D.M., Fahey T.J., Fernandez C.W., Guo D., Helmisaari H.S., Hobbie E.A., Iversen C.M., Jackson R.B. (2015). Redefining fine roots improves understanding of below-ground contributions to terrestrial biosphere processes. New Phytol..

[34] Koevoets I.T., Venema J.H., Elzenga J.T., Testerink C. (2016). Roots Withstanding their Environment: Exploiting Root System Architecture Responses to Abiotic Stress to Improve Crop Tolerance. Front. Plant Sci..

[35] The Tomato Genome Consortium (2012). The tomato genome sequence provides insights into fleshy fruit evolution. Nature.

[36] Fernandez-Pozo N., Menda N., Edwards J.D., Saha S., Tecle I.Y., Strickler S.R., Bombarely A., Fisher-York T., Pujar A., Foerster H. (2015). The Sol Genomics Network (SGN)—From genotype to phenotype to breeding. Nucleic Acids Res..

[37] Potter S.C., Luciani A., Eddy S.R., Park Y., Lopez R., Finn R.D. (2018). HMMER web server: 2018 update. Nucleic Acids Res..

[38] Mistry J., Chuguransky S., Williams L., Qureshi M., Salazar G.A., Sonnhammer E.L., Tosatto S.C., Paladin L., Raj S., Richardson L.J. (2021). Pfam: The protein families database in 2021. Nucleic Acids Res..

[39] Wilkins M.R., Gasteiger E., Bairoch A., Sanchez J.C., Williams K.L., Appel R.D., Hochstrasser D.F. (1999). Protein identification and analysis tools in the ExPASy server. Methods Mol. Biol..

[40] Chen C., Chen H., Zhang Y., Thomas H.R., Frank M.H., He Y., Xia R. (2020). TBtools: An Integrative Toolkit Developed for Interactive Analyses of Big Biological Data. Mol. Plant.

[41] Ma W., Liu X., Chen K., Yu X., Ji D. (2023). Genome-Wide Re-Identification and Analysis of CrRLK1Ls in Tomato. Int. J. Mol. Sci..

[42] Wang Y., Zhang J., Hu Z., Guo X., Tian S., Chen G. (2019). Genome-Wide Analysis of the MADS-Box Transcription Factor Family in Solanum lycopersicum. Int. J. Mol. Sci..

[43] Tamura K., Dudley J., Nei M., Kumar S. (2007). MEGA4: Molecular Evolutionary Genetics Analysis (MEGA) software version 4.0. Mol. Biol. Evol..

[44] Lescot M., Déhais P., Thijs G., Marchal K., Moreau Y., Van de Peer Y., Rouzé P., Rombauts S. (2002). PlantCARE, a database of plant cis-acting regulatory elements and a portal to tools for in sillco analysis of promoter sequences. Nucleic Acids Res..

[45] Chow C.-N., Lee T.-Y., Hung Y.-C., Li G.-Z., Tseng K.-C., Liu Y.-H., Kuo P.-L., Zheng H.-Q., Chang W.-C. (2019). PlantPAN3.0: A new and updated resource for reconstructing transcriptional regulatory networks from ChIP-seq experiments in plants. Nucleic Acids Res..

[46] Szklarczyk D., Gable A.L., Nastou K.C., Lyon D., Kirsch R., Pyysalo S., Doncheva N.T., Legeay M., Fang T., Bork P. (2021). The STRING database in 2021: Customizable protein–protein networks, and functional characterization of user-uploaded gene/measurement sets. Nucleic Acids Res..

[47] Lopes C.T., Franz M., Kazi F., Donaldson S.L., Morris Q., Bader G.D. (2010). Cytoscape Web: An interactive web-based network browser. Bioinformatics.

[48] Ai G., Zhang D., Huang R., Zhang S., Li W., Ahiakpa J.K., Zhang J. (2020). Genome-Wide Identification and Molecular Characterization of the Growth-Regulating Factors-Interacting Factor Gene Family in Tomato. Genes.

[49] Wittwer C.T., Vandesompele J., Shipley G.L., Pfaffl M.W., Nolan T., Mueller R., Kubista M., Huggett J., Hellemans J., Garson J.A. (2009). The MIQE Guidelines: Minimum Information for Publication of Quantitative Real-Time PCR Experiments. Clin. Chem..

[50] Li G., Wang J., Zhang C., Ai G., Zhang D., Wei J., Cai L., Li C., Zhu W., Larkin R.M. (2021). L2, a chloroplast metalloproteinase, regulates fruit ripening by participating in ethylene autocatalysis under the control of ethylene response factors. J. Exp. Bot..

