# Supplementary material for: Genome-Wide Analysis of the DC1 Domain Protein Gene Family in Tomatoes under Abiotic Stress
Source: Int J Mol Sci. 2023 Nov 30;24(23):16994. doi: 10.3390/ijms242316994 (PMC10707348; doi:10.3390/ijms242316994)
Supplement: Supplementary file 1 [file ijms-24-16994-s001.zip › ijms-2678969-supplementary.pdf]

Table S1. Primers used in the study of *SlCHP* relative expression

primers	Sequences(5'-3')	Experiments
SlCHP1-Q-FW	GCTCCAATGCATGCCTCGTCT	qRT-PCR
SlCHP1-Q-RV	CCACCTTGAGGAGGGTATGGC	
SlCHP2-Q-FW	CAGTAGAGAAACCAGCAGACAAATCAG	
SlCHP2-Q-RV	CGACTCCTCTTCTTCTCCTCCTC	
SlCHP3-Q-FW	GGGAAGACAGAATAAGAGTAGCAATGCG	
SlCHP3-Q-RV	CTGTTGCATCCACGGAAATTAGGC	
SlCHP4-Q-FW	ATGGGAAGACTGAACGGGGGTAC	
SlCHP4-Q-RV	CGCGGAGCATTAAAACAGTTTTCGT	
SlCHP5-Q-FW	CAGCAGCGTCCAGTTCCAAATC	
SlCHP5-Q-RV	GTAACCGTATCGTCTTCGTGGAGTC	
SlCHP6-Q-FW	GAAGAGGCAGAGGAGGAAGAAGC	
SlCHP6-Q-RV	GTAAGGAGAACCAAAGCAGAGCTCC	
SlCHP7-Q-FW	CCCCTTATGATGATGACGCCGATG	
SlCHP7-Q-RV	CCTTCTCTGTTTTCCTCTGTTTCGGTTC	
SlCHP8-Q-FW	TCCCCCTTCACAAATCATGCTTCG	
SlCHP8-Q-RV	TCAGGCCAAGAGGTTGCACATTTAG	
SlCHP9-Q-FW	AGGCACACATTTACAATGCGCAAC	
SlCHP9-Q-RV	TAAGGAGGATGAGAGCTACGCCAC	
SlCHP10-Q-FW	CAAGTCATTTGCTCGGGCTGTG	
SlCHP10-Q-RV	CGGAGACTTAGAGCGTGTTTCGG	
SlCHP11-Q-FW	CTGCTTCCAAAGCCCATTTACCCC	
SlCHP11-Q-RV	GGTGACAATGAGACCGATGAGCG	
SlCHP12-Q-FW	ACATATGCATTGTGCAATTCCTTCACC	
SlCHP12-Q-RV	CCATTTATGTCCTTCTCACAAGCGTTG	
SlCHP13-Q-FW	CGTACGGATGCAAACGTTGTAGGTA	
SlCHP13-Q-RV	CAGGATTATCACCAACGTGACATGCG	
SlCHP14-Q-FW	GTTCACATAACCAGTTCCAGCAG	
SlCHP14-Q-RV	CTGATTTGGCATTGCTTGTCCTG	

SlCHP15-Q-FW	CCTGCTCAGGTTGCAAGGAGTTT	
SlCHP15-Q-RV	CTTGGCCACGCTATTCCAGCT	
SlCHP16-Q-FW	TGTCCTGCTTGTGAGTTTGATGTTC	
SlCHP16-Q-RV	AATAGCCTGCTGGAATTGGTTAGTG	
SlCHP17-Q-FW	ATGCAGCAACAACCCCCATCC	
SlCHP17-Q-RV	CAAAGGCTGAGCTAAATCAGACGCT	
SlCHP18-Q-FW	GAAGGGGCAGCCGTCTTATGTTC	
SlCHP18-Q-RV	GGAGGAGCAACGATTTCTGTACTTCAG	
SlCHP19-Q-FW	ATGGCACCTATCCCAACAACCATTC	
SlCHP19-Q-RV	TGAGTGGGTGGTGAGGATGCAT	
SlCHP20-Q-FW	ATGGCACCTATCCACAAGAACCCAA	
SlCHP20-Q-RV	GGACATGTTCCACAATATTCGTGTAGATCG	
SlCHP21-Q-FW	GCCATCCACATGATCTTCGTACCAT	
SlCHP21-Q-RV	CGGATAGAGAAGGCGTAAGGGGTG	
SlCHP11-2300-FW	cgggggacgagctcggtaccATGGGGAAGACGAAGTTAGAACCT	Subcellular Localization
SlCHP11-2300-RV	accatggtgtcgactctagaGAATTTAAGATTTCCAAACAATGAATTC	
SlCHP13-2300-FW	cgggggacgagctcggtaccATGGACTCCCATTTGAAAATCAA	
SlCHP13-2300-RV	accatggtgtcgactctagaCTCAACCTCTAGATCGCGTAGC	
SlCHP14-2300-FW	cgggggacgagctcggtaccATGGGGAGGTTCAAGTTGAATGG	
SlCHP14-2300-RV	accatggtgtcgactctagaGTTCTGCGACGAGTCACCAAA	
SlCHP15-2300-FW	cgggggacgagctcggtaccATGATGAACAGCACCAGTATAAAACC	
SlCHP15-2300-RV	accatggtgtcgactctagaTGCAATTCTTCTTCTGTTAGTGCTT	
SlCHP17-2300-FW	cgggggacgagctcggtaccATGCAGCAACAACCCCCATC	
SlCHP17-2300-RV	accatggtgtcgactctagaTGAATCTTTTCGATCCTCAGAAGC	
SlCHP18-2300-FW	cgggggacgagctcggtaccATGCCGGACGACAAGAAGATG	
SlCHP18-2300-RV	accatggtgtcgactctagaCTTGGAGTGGTCTTCCTCTATT	

SlCHP20-2300-FW	cgggggacgagctcggtaccATGGCACCTATCCACAAGAACC
SlCHP20-2300-RV	accatggtgtcgactctagaCAGCCCAAGAATCAACGCAG
